# Deep learning-based multi-frequency denoising for myocardial perfusion SPECT

**DOI:** 10.1186/s40658-024-00680-w

**Published:** 2024-10-02

**Authors:** Yu Du, Jingzhang Sun, Chien-Ying Li, Bang-Hung Yang, Tung-Hsin Wu, Greta S. P. Mok

**Affiliations:** 1 Biomedical Imaging Laboratory (BIG), Department of Electrical and Computer Engineering, Faculty of Science and Technology, University of Macau, Taipa, Macau SAR China; 2 Center for Cognitive and Brain Sciences, Institute of Collaborative Innovation, University of Macau, Taipa, Macau SAR China; 3 School of Cyberspace Security, Hainan University, Haikou, Hainan China; 4 Department of Biomedical Imaging and Radiological Sciences, National Yang Ming Chiao Tung University, Taipei, Taiwan; 5 Department of Nuclear Medicine, Taipei Veterans General Hospital, Taipei, Taiwan

**Keywords:** Deep learning, Myocardial perfusion SPECT, Generative adversarial network, Denoising

## Abstract

**Background:**

Deep learning (DL)-based denoising has been proven to improve image quality and quantitation accuracy of low dose (LD) SPECT. However, conventional DL-based methods used SPECT images with mixed frequency components. This work aims to develop an integrated multi-frequency denoising network to further enhance LD myocardial perfusion (MP) SPECT denoising.

**Methods:**

Fifty anonymized patients who underwent routine ^99m^Tc-sestamibi stress SPECT/CT scans were retrospectively recruited. Three LD datasets were obtained by reducing the 10 s acquisition time of full dose (FD) SPECT to be 5, 2 and 1 s per projection based on the list mode data for a total of 60 projections. FD and LD projections were Fourier transformed to magnitude and phase images, which were then separated into two or three frequency bands. Each frequency band was then inversed Fourier transformed back to the image domain. We proposed a 3D integrated attention-guided multi-frequency conditional generative adversarial network (AttMFGAN) and compared with AttGAN, and separate AttGAN for multi-frequency bands denoising (AttGAN-MF).The multi-frequency FD and LD projections of 35, 5 and 10 patients were paired for training, validation and testing. The LD projections to be tested were separated to multi-frequency components and input to corresponding networks to get the denoised components, which were summed to get the final denoised projections. Voxel-based error indices were measured on the cardiac region on the reconstructed images. The perfusion defect size (PDS) was also analyzed.

**Results:**

AttGAN-MF and AttMFGAN have superior performance on all physical and clinical indices as compared to conventional AttGAN. The integrated AttMFGAN is better than AttGAN-MF. Multi-frequency denoising with two frequency bands have generally better results than corresponding three-frequency bands methods.

**Conclusions:**

AttGAN-MF and AttMFGAN are promising to further improve LD MP SPECT denoising.

**Supplementary Information:**

The online version contains supplementary material available at 10.1186/s40658-024-00680-w.

## Introduction

Myocardial perfusion single photon emission computed tomography (MP SPECT) is a well-established non-invasive method for cardiovascular disease [1]. However, a relatively long acquisition time (15–20 min for NaI SPECT [2] ) and high radioactive tracer injection are needed to obtain sufficient photon counts and high image quality MP SPECT, which lead to patients’ discomfort, potential mismatch with fast helical CT scans [3–5] and radiation exposure concern [6]. Though dedicated cardiac scanners with CZT detectors [7] can substantially reduce scan time (3–6 min) [8], it is still much longer than that of a CT scan.

Low dose (LD) and fast MP SPECT is desirable yet Poisson noise would increase as detected photon counts decrease, degrading quantification accuracy, image quality and subsequent clinical diagnosis. Deep learning (DL)-based methods have been proven to be promising for denoising LD MP SPECT. Previously, Shiri et al. [9] developed a 2D residual convolutional neural network (ResNet) to denoise MP SPECT projections, generating full dose (FD) projections from LD projections with 1/2 acquisition time or 1/2 projection number. They concluded that DL-based methods were feasible to recover the quantification errors introduced by reduced acquisition time and projection number. Ramon et al. [10] used a 3D convolutional neural network (CNN) to denoise LD MP SPECT on reconstruction-domain and demonstrated that DL denoised 1/2 LD SPECT can achieve same diagnostic accuracy as the FD SPECT. In addition, they pooled 1/2, 1/4, 1/8, and 1/16 LD SPECT together for “one-size-fits-all” training and found that it has inferior results than dose level-specific training. Liu et al. [11] developed a 3D U-Net-based denoising method where the 1/4 LD and 1/8 LD datasets were combined for training, which outperformed dose-specific denoising in detecting perfusion defects at both 1/4 and 1/8 dose levels. These results might come from the fact that 1/4 vs. 1/8 LD SPECT were more similar as compared to 1/2 vs. 1/16 LD SPECT. Aghakhan et al. [12] used a 2D conditional generative adversarial network (cGAN) to denoise LD MP SPECT projections and found that the injection dose can be reduced down to 1/4, while lower dose levels are not feasible due to the very poor signal-to-noise ratio and huge errors in total perfusion deficit (TPD) analysis. Sohlberg et al. [13, 14] showed improved noise performance and slightly lower perfusion defect detection performance when using 3D cGAN than FD MP SPECT on 1/2 and 1/4 LD levels based on a receiver-operating-characteristic (ROC) study. Chen et al. [15] proposed a cascaded Dual-Domain Coarse-to-Fine Progressive Network for simultaneous LD denoising, limited view reconstruction, and CT-free µ-map generation of cardiac SPECT and achieved superior performance to existing single- or multi-task networks under difference LD levels up to 1/10 and various iterations. Recently, our group implemented a 3D cGAN to denoise dual cardiac and respiratory gating MP SPECT [16] and static MP SPECT on projection- and reconstruction-domain [17]. We demonstrated that denoising on the former is superior to the later. Our group then developed and compared a 3D attention-guided cGAN (AttGAN) with U-Net and cGAN, showing that AttGAN has improved denoising performance than the others [18]. A recent review [19] on low dose emission tomography denoising summaries supervised DL-based denoising methods [12, 16–18, 20] for MP SPECT.

However, all the conventional DL-based denoising methods were performed on SPECT images with mixed frequency components, where the lower frequency component mainly represents the image background, mid-frequency component represents image details and high frequency component mainly represents noise. We have preliminary evaluated its performance with standard AttGAN denoising and multi-frequency denoising by AttGANs [21] with multiple frequency components (AttGAN-MF) on clinical MP SPECT data. In this study, we further proposed an attention-guided multi-frequency generative adversarial network (AttMFGAN), which denoised different frequency components of the projection images separately in different generators with 1 integrated discriminator for MP SPECT.

## Materials and methods

### Clinical dataset

A population of 50 anonymized patients (Table 1) who underwent routine stress SPECT/CT scan on a CZT SPECT/CT system (NM/CT 870 CZT, GE Healthcare, USA) were retrospectively recruited in this study under the local ethics approval (IRB number 2022-11-002CC). Sixty projections were acquired through 180° from right anterior oblique to left posterior oblique with an acquisition time of 10 s/view for FD projections, after 1184 MBq ^99m^Tc-sestamibi injection. The LD projections were obtained by reducing the 10 s/view acquisition time to 5 s/view, 2 s/view and 1 s/view based on the list-mode data of FD projections. A helical CT was scanned in the chest region prior to the SPECT scan, with 120 kVp, smart tube current (10–150 mA) and 0.375 cm thickness. The reconstructed CT scans were resampled to the matrix size (64 × 64 × 64) and voxel size (0.6096 cm) of SPECT images and registered to SPECT for attenuation corrections (AC). The 3D OS-EM algorithm was used to reconstruct the SPECT projections, running up to 5 iterations and 4 subsets with dual energy window scatter correction [22] and CT-based AC. The reconstructed FD SPECT were then filtered with a Gaussian filter with standard deviation of 0.8 voxel.

**Table 1:** Demographic information for the patient study

	Male	Female	Total
Number	37	13	50
Age (yr)	69.2 ± 9.73 (56–90)	64.9 ± 11.24 (42–83)	66.0 ± 10.94 (42–90)
BMI (kg/m^2^)	25.0 ± 2.65 (17.91–30.11)	24.5 ± 3.13 (21.09–30.47)	25.0 ± 2.92 (17.92–31.60)

### Projections in multi-frequency bands

**Fig. 1:**
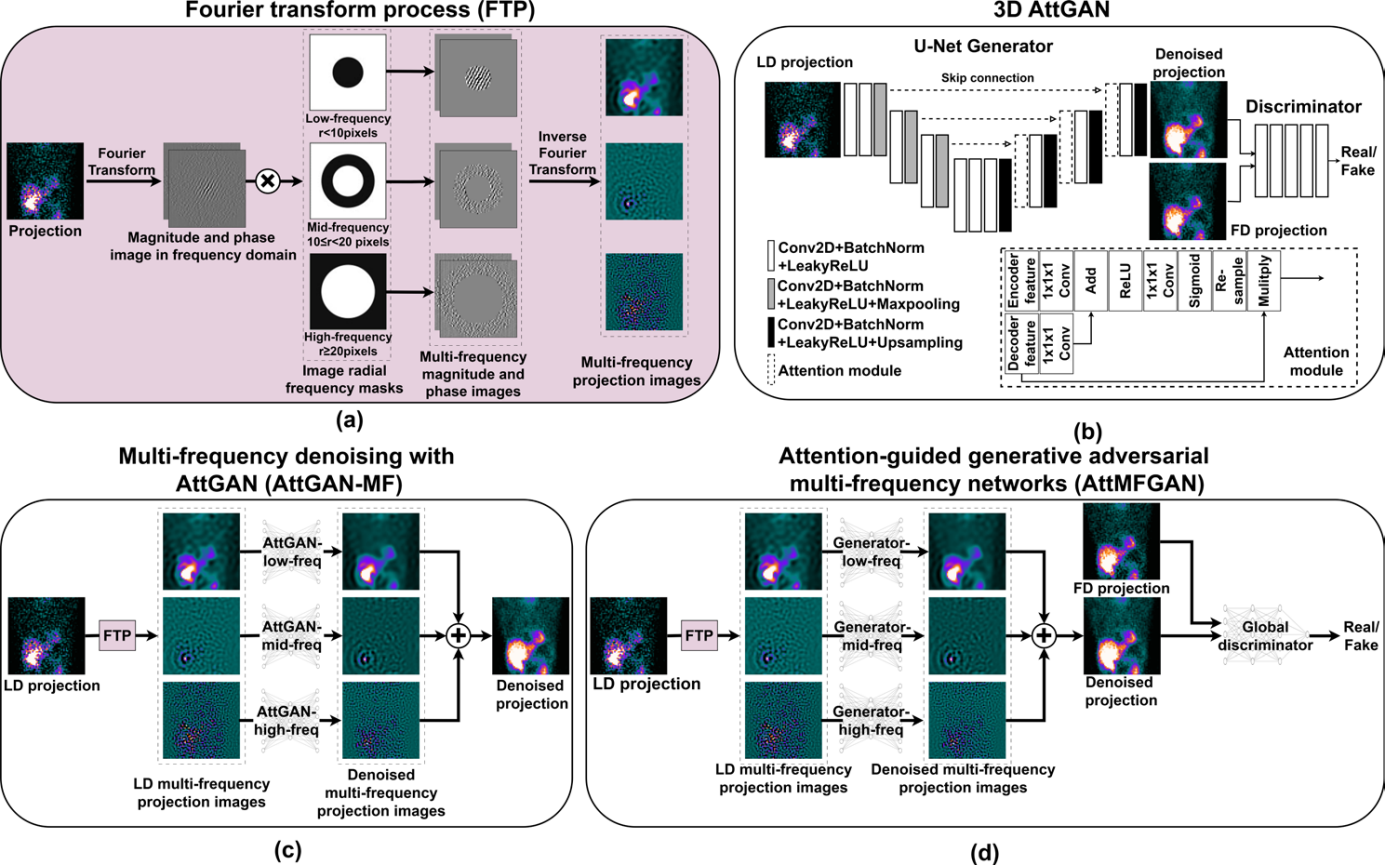
(**a**) Multi-frequency projections generation based on Fourier transform. (**b**) Conventional 3D AttGAN. (**c**) Multi-frequency denoising using multiple AttGANs (AttGAN-MF). (**d**) An integrated AttGAN for multi-frequency denoising (AttMFGAN). Here M = 3

The SPECT projections were Fourier transformed to magnitude and phase images in the frequency domain using 2D Fast Fourier Transform (FFT) algorithm [23] for each projection view. The magnitude image and phase image were then separated by radial frequency masks with different radii centered at the image center into two or three bands (Fig. 1(a)). Here we used a 10-voxel radius mask for low- and high- frequency separation for 2 frequency bands, and an additional 20-voxel radius mask to separate the mid- and high-frequency for 3 frequency bands. The magnitude and phase images within the same frequency band were then inverse Fourier transformed back to the image domain to generate SPECT projections in multi-frequency (MF, M = 2/3) bands.

### Multi-frequency denoising

We implemented a 3D AttGAN [18] (Fig. 1(b)) by adding attention blocks in a 3D cGAN [24, 25] as our baseline denoising method. The objective function for 3D AttGAN is:

1 $$\eqalign{{L_{AttGAN}}{\rm{ }} = {\rm{ }} & BCE{\rm{ }}(D{\rm{ }}(G(LD))){\rm{ }} \cr & + \lambda MAE{\rm{ }}(G(LD){\rm{ }},{\rm{ }}FD) \cr}$$

where BCE was the binary cross entropy loss for the discriminator (*D*). MAE was the mean absolute error loss for the generator (*G*). $\:\lambda\:$ was the weight to balance the loss of generator and discriminator, and $\:\lambda\:=20$ was used in this study [24].

Then, the multi-frequency projection images were denoised by two or three AttGANs (AttGAN-MF, M = 2/3; Fig. 1(c)) separately. The denoised multi-frequency projections images were then added together to form the final denoised projection. The overall objective function of AttGAN-MF for frequency band $\:f$ could be expressed as:

2 $$\eqalign{{{\rm{L}}_{{\rm{AttGAN{\rm{ - M}}{{\rm{F}}_{\rm{f}}}}}}}{\rm{ = }} & {\rm{BCE (}}{{\rm{D}}_{\rm{f}}}{\rm{ (}}{{\rm{G}}_{\rm{f}}}{\rm{ (L}}{{\rm{D}}_{{\rm{f }}}}{\rm{))) }} \cr & {\rm{ + }}\lambda {\rm{MAE (}}{{\rm{G}}_{\rm{f}}}{\rm{ (L}}{{\rm{D}}_{\rm{f}}}{\rm{ ) , F}}{{\rm{D}}_{\rm{f}}}{\rm{ ) , f }} \in {\rm{ F}} \cr}$$

where *F=*{*low-freq*,* high-freq*} or {*low-freq*,* mid-freq*,* high-freq*} frequency bands. $\:{LD}_{f}$ and $\:{FD}_{f}$ denoted multi-frequency LD and FD projections respectively.

We proposed to use multiple generators to denoise multi-frequency LD SPECT projection images, which were then added to form the denoised LD SPECT projections as one input to the discriminator (AttMFGAN, M = 2/3; Fig. 1(d)). The global discriminator would discriminate the summed denoised LD projections ($\:\sum\:_{f\in\:F}{G}_{f}\left({LD}_{f}\right)$) from the generators and corresponding real FD projections using BCE loss. The objective function of generators in AttMFGAN for frequency band $\:f$ can be expressed as:

3 $$\eqalign{{L_{AttMFGAN}}_{_f}{\rm{ }} & = \\&{\rm{ }}BCE(D(\sum _{f{\rm{ }}{ \in F}}{G_f}{\rm{ }}(L{D_f}{\rm{ }})) \cr \\& + {\rm{ }}{\lambda _1}MAE{\rm{ }}({G_f}{\rm{ }}(L{D_f}{\rm{ }}),{\rm{ }}F{D_f}){\rm{ }} \cr \\& + {\rm{ }}{\lambda _2}MAE({\sum _{f{\rm{ }} \in F}}{G_f}{\rm{ }}(L{D_f}{\rm{ }}),{\rm{ }}FD),{\rm{ }}f{\rm{ }} \in {\rm{ }}F \cr}$$

where $\:{\lambda\:}_{1}={\lambda\:}_{2}=10$. The first MAE was the local loss between denoised $\:({G}_{f}\left({LD}_{f}\right))$ and $\:{FD}_{f}$ in different frequency bands, while the second MAE was the global loss between final denoised SPECT projection ($\:\sum\:_{f\in\:F}{G}_{f}\left({LD}_{f}\right)$) and the original FD projections. As an ablation study, we investigated the potential performance improvement from the use of global MAE on denoising 1/10 LD images for Att2FGAN versus just using the local MAE (Att2FGAN-L) or global MAE (Att2FGAN-G):

4 $$\eqalign{{{\rm{L}}_{{\rm{Att2FGAN{\rm{ - L}}{_{\rm{f}}}}}}} & = BCE(D(\mathop \sum \limits_{f \in F} {G_f}(LDf)) \cr & + \lambda \,MAE({G_f}(L{D_f}),F{D_f}),f \in F \cr}$$

5 $$\eqalign{{\rm{L}}_{{\rm{Att2FGAN{\rm{ - G}}}}} & = {\rm{ }}BCE(D({\sum _{f \in {\rm{ }}F}}{G_f}{\rm{ }}(L{D_{f{\rm{ }}}}))) \cr & + \lambda \,MAE({\sum _{f \in {\rm{ }}F}}{G_f}{\rm{ }}(L{D_f}{\rm{ }}),{\rm{ }}FD),{\rm{ }}f{\rm{ }} \in {\rm{ }}F \cr}$$

where $\:\lambda\:=20$, *F=*{*low-freq*,* high-freq*} here.

### Network training

The paired whole volumes of LD and FD MP SPECT projections in different frequency bands of 50 patients were divided into 35, 5, and 10 for training, validation and testing. A 5-fold cross-validation was applied to test all 50 patients. After the training, the testing LD MP SPECT projections were input to the trained model to get denoised projections in different frequency bands. The denoised projections in different frequency bands were combined to get the denoised projections, which were then reconstructed with the same OS-EM algorithm as the FD projections. No further post-reconstruction filter was applied on the DL-denoised images.

The hyperparameters of all networks were set to be 3 layers of encoder-decoder depth for the U-Net-based generator and 32 feature map numbers, based on our previous work [17]. Learning rate decay was applied with an initial learning rate of 0.001. All networks were implemented using the Tensorflow framework and trained on a NVIDIA RTX 4090 GPU. The training time was ~ 2 h for AttGAN, ~ 4.5 h for AttGAN-2 F/Att2FGAN, and ~ 6.5 h for AttGAN-3 F/Att3FGAN.

A bilateral filter [26] was implemented as a conventional post-reconstruction filter-based denoising approach using OpenCV package (Ver. 4.10.0) in Python as baseline comparison:

6 $$\:\stackrel{\sim}{I}\left(x\right)=\frac{1}{C}\sum\:_{y\in\:N\left(x\right)}{e}^{\frac{-{||y-x||}^{2}}{2{\sigma\:}_{d}^{2}}}{e}^{\frac{-{\left|I\left(y\right)-I\left(x\right)\right|}^{2}}{2{\sigma\:}_{r}^{2}}}I\left(y\right)$$

where *I(x)*, *I(y)* are image intensity at pixel *x* and *y*, *σ*_*d*_ and *σ*_*r*_ are parameters controlling the fall-off of the weights for spatial and intensity distances, respectively, *N(x)* is a spatial neighborhood of *x* and *C* is the normalization constant.

7 $$\:C=\sum\:_{y\in\:N\left(x\right)}{e}^{\frac{-{||y-x||}^{2}}{2{\sigma\:}_{d}^{2}}}{e}^{\frac{-{\left|I\left(y\right)-I\left(x\right)\right|}^{2}}{2{\sigma\:}_{r}^{2}}}$$

The parameters of pixel neighborhood, filter sigma for spatial distance and the intensity distance were optimized to be 5, 8 and 2 voxels based on average NMSE of bilateral filtered 1/10 LD reconstructed SPECT and FD reconstructed SPECT of the 50 patients (Supplementary Table S1).

### Data analysis

The normalized mean square error (NMSE), structural similarity (SSIM), peak signal-to-noise ratio (PSNR), joint histogram and linear regression were assessed on a 3D cardiac volume-of-interest (VOI, 20 × 20 × 20, supplementary Figure S1 (a)) of the reconstructed SPECT images. Different denoising methods were compared to the Gaussian filtered FD SPECT as reference. The 17-segment analysis was also performed on the polar plots.

For the clinical-relevant index, we used the Wackers-Liu™ (WLCQ) software (Voxelon Inc, Watertown, CT) [27] to measure the perfusion defect size (PDS, %LV) on the reconstructed SPECT images. The MAE on PDS was measured between denoised and FD SPECT images.

The coefficient of variance (CoV), used as the noise index, was calculated based on a 3D uniform volume-of-interest (6 × 6 × 3 voxels) in lungs (supplementary Figure S1 (a)) based on 1/10 LD SPECT images by different denoising methods. The NMSE-CoV and SSIM-CoV trade-off curves were then plotted with different OS-EM reconstruction update numbers.

8 $$\:CoV=\frac{\sqrt{\frac{1}{m-1}\sum\:_{i=1}^{m}{({x}_{i}-\stackrel{-}{x})}^{2}}}{\stackrel{-}{x}}$$

where $\:m$ is the total voxel number in the 3D VOI, $\:{x}_{i}$ is the intensity of voxel $\:i$, and $\:\stackrel{-}{x}$ is the average intensity in the 3D VOI.

A paired *t*-test [12] (SPSS, IBM Corporation, Armonk, NY, USA) was applied to NMSE, SSIM, PSNR, and MAE on PDS for statistical analysis.

## Results

**Fig. 2:**
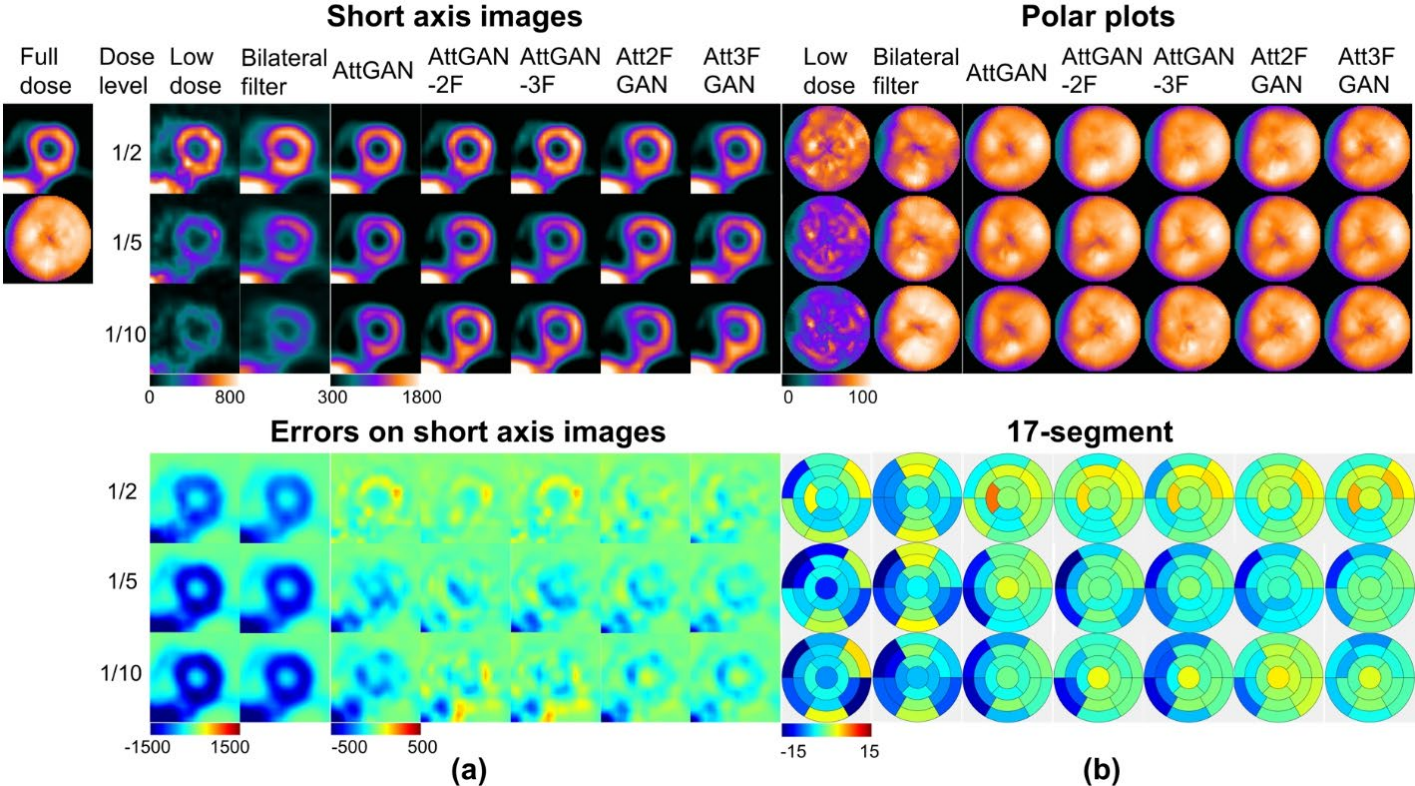
(**a**) Sample short axis images of different denoising methods (top) and corresponding error maps (bottom) compared to full dose images of a male normal patient (BMI = 19.2 kg/m^2^, age = 76 years old). (**b**) Corresponding polar plots of different denoising methods (top) and 17-segment errors compared to those of the full dose images for the same patient (bottom)

The results of one normal patient and one patient with cardiac defect located at septum are shown in Fig. 2. AttGAN-MF shows less errors on the myocardium region of short-axis (SA) or horizontal long axis (HLA) images as compared to AttGAN, while AttMFGAN shows further improved denoising performance with less errors on SA images than AttGAN-MF. For polar plots, the proposed AttMFGAN has generally less bias from 17-segment analysis than AttGAN-MF, followed by conventional AttGAN for both patients.

The average NMSE, SSIM, PSNR and MAE on PDS and corresponding 95% confidence interval (CI) of all 50 patients are summarized in Table 2. For voxel-based bias assessment, on 1/10 LD level, the NMSE values are 0.1222 (95% CI 0.0985–0.1459), 0.0620 (0.0511–0.0729), 0.0527 (0.0441–0.0613), 0.0618 (0.0503–0.0733), 0.0441 (0.0353–0.0529), 0.0522 (0.0436–0.0608) for bilateral filter, AttGAN, AttGAN-2 F, AttGAN-3 F, Att2FGAN and Att3FGAN respectively. All methods have significantly lower NMSE as compared to LD SPECT (all *p* < 0.001). All DL-based methods are significantly better than bilateral filter on NMSE (all *p* < 0.01). AttGAN-MF has a lower NMSE as compared to AttGAN (*p* < 0.05), while the NMSE values are further lowered by AttMFGAN (*p* < 0.05) compared to AttGAN-MF. The SSIM and PSNR results follow the same trend as NMSE for all methods. For MAE on PDS, all DL denoising methods are all significantly better than bilateral filter, while the proposed Att2FGAN has the best quantification results among all DL methods, i.e., 7.77 (95% CI 6.1–9.44), 3.40 (2.31–4.49), 2.36 (1.74–2.98), 3.18 (2.19–4.17), 1.91 (1.51–2.31), 2.88 (2.11–3.65) for bilateral filter, AttGAN, AttGAN-2 F, AttGAN-3 F, Att2FGAN and Att3FGAN on 1/10 LD level respectively. Results for different dose levels are consistent.

**Table 2:** The NMSE, SSIM, PSNR, and MAE on PDS measurements (MEAN (95% CI)) on the cardiac VOI of the reconstructed MP SPECT images using different denoising methods on 50 tested patients. Bold values indicate best results. The significance of paired t-test between each method and Att2FGAN is marked by asterisk. (↓: lower value is better, ↑: higher value is better; *: *p* < 0.05, **: *p* < 0.01, ***: *p* < 0.001)

LD level	Method	NMSE ↓	SSIM ↑	PSNR ↑	MAE on PDS ↓
1/2	LD	0.0866 (0.0798–0.0934)***	0.8023 (0.7924–0.8122)***	16.18 (15.85–16.51)***	3.1 (2.21–3.99)***
	Bilateral filter	0.0633 (0.0575–0.0691)***	0.8222 (0.8114–0.833)***	17.45 (17.09–17.81)***	2.84 (2.03–3.65)***
	AttGAN	0.0300 (0.0284–0.0316)*	0.8661 (0.8579–0.8743)*	28.53 (27.81–29.25)**	0.71 (0.40–1.02)**
	AttGAN-2 F	0.0267 (0.0247–0.0287)*	0.873 (0.8629–0.8831)*	28.59 (28.16–29.02)**	0.4 (0.25–0.55)**
	AttGAN-3 F	0.0274 (0.0246–0.0302)*	0.886 (0.8751–0.8969)	28.61 (28.12–29.10)*	0.49 (0.36–0.62)*
	Att2FGAN	**0.0229 (0.0206–0.0252)**	**0.8876 (0.8821–0.8931)**	**29.86 (29.51–30.21)**	**0.38 (0.26–0.5)**
	Att3FGAN	0.0235 (0.0212–0.0258)	0.8801 (0.8724–0.8878)	29.38 (29.04–29.72)	0.45 (0.27–0.63)*
1/5	LD	0.1986 (0.184–0.2132)***	0.6503 (0.6341–0.6665)***	13.6 (13.27–13.93)***	6.74 (5.53–7.95)***
	Bilateral filter	0.1000 (0.0891–0.1109)***	0.7355 (0.7224–0.7486)**	17.1 (16.67–17.53)***	4.93 (3.87–5.99) ***
	AttGAN	0.0506 (0.0419–0.0593)**	0.8227 (0.8071–0.8383)**	26.73 (26.27–27.19)**	1.85 (1.31–2.39)*
	AttGAN-2 F	0.0390 (0.0356–0.0424)*	0.8418 (0.836–0.8476)	27.26 (26.89–27.63)*	0.88 (0.67–1.09)*
	AttGAN-3 F	0.0413 (0.0381–0.0445)*	0.8293 (0.8151–0.8435)**	27.09 (26.69–27.49)	1.36 (0.96–1.76)**
	Att2FGAN	**0.0381 (0.0346–0.0416)**	**0.8443 (0.8360–0.8526)**	**28.93 (28.44–29.42)**	**0.61 (0.45–0.77)**
	Att3FGAN	0.0411 (0.0372–0.045)*	0.8378 (0.8307–0.8449)*	28.38 (27.81–28.95)	1.02 (0.75–1.29)*
1/10	LD	0.2919 (0.2691–0.3147)***	0.5468 (0.5235–0.5701)	12.91 (12.59–13.23)***	9.84 (8-11.68)***
	Bilateral filter	0.1222 (0.0985–0.1459)***	0.6911 (0.6707–0.7115) **	14.55 (13.64–15.46)***	7.77 (6.1–9.44)***
	AttGAN	0.0620 (0.0511–0.0729)**	0.7842 (0.7695–0.7989)*	24.43 (23.83–25.03)**	3.4 (2.31–4.49)**
	AttGAN-2 F	0.0527 (0.0441–0.0613)**	0.787 (0.7712–0.8028)*	26.73 (25.89–27.57)**	2.36 (1.74–2.98)*
	AttGAN-3 F	0.0618 (0.0503–0.0733)**	0.7845 (0.7677–0.8013)	25.66 (24.96–26.36)**	3.18 (2.19–4.17)*
	Att2FGAN	**0.0441 (0.0353–0.0529)**	**0.7978 (0.7836–0.8120)**	**27.13 (26.73–27.53)**	**1.91 (1.51–2.31)**
	Att3FGAN	0.0522 (0.0436–0.0608)*	0.7825 (0.7698–0.7952)*	26.46 (25.96–26.96)*	2.88 (2.11–3.65)*

**Fig. 3:**
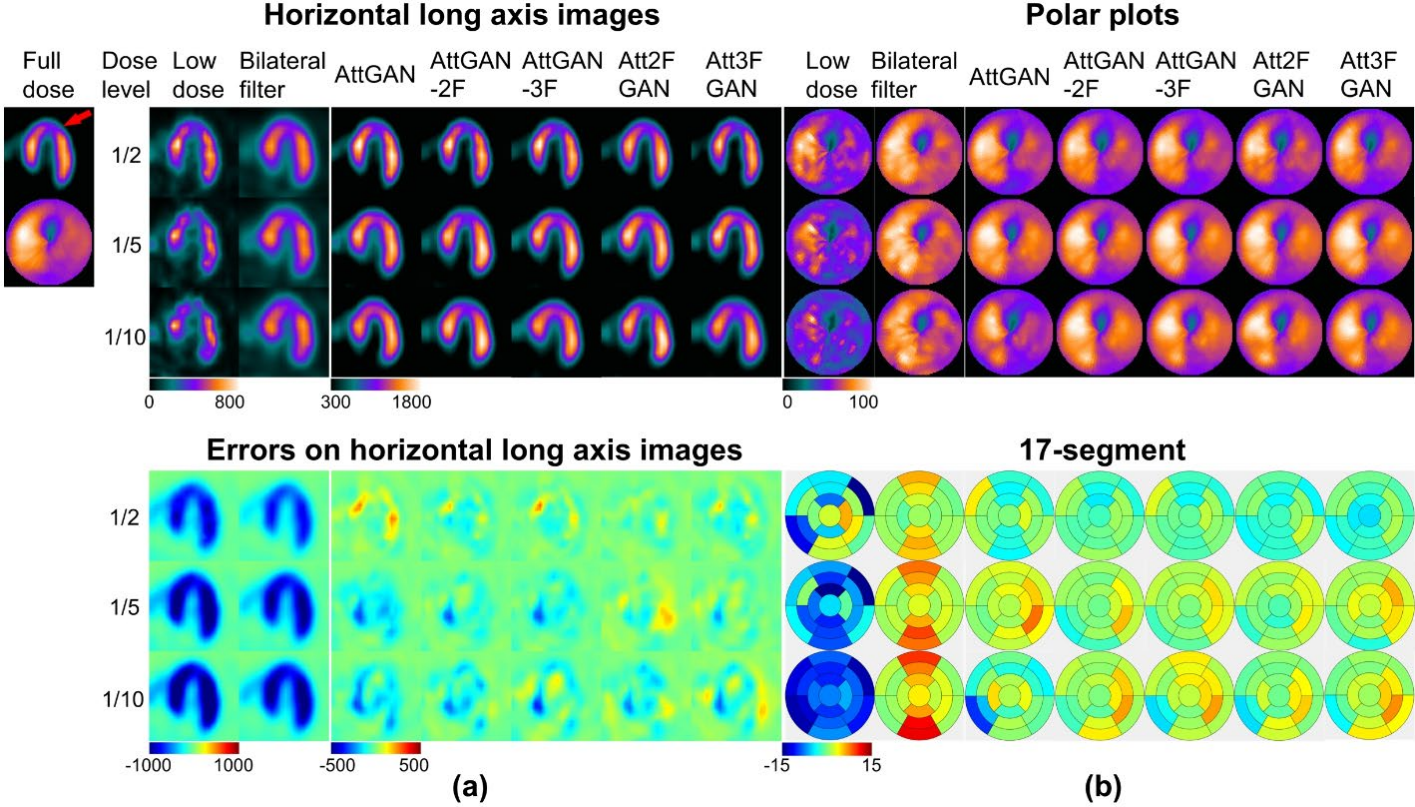
(**a**) Sample horizontal long axis images of different denoising methods (top) and corresponding error maps (bottom) compared to full dose images of a male patient (BMI = 24.2 kg/m^2^, age = 81 years old) with a cardiac defect on the septum (red arrow). (**b**) Corresponding polar plots of different denoising methods (top) and 17-segment errors compared to those of the full dose images for the same patient (bottom)

**Fig. 4:**
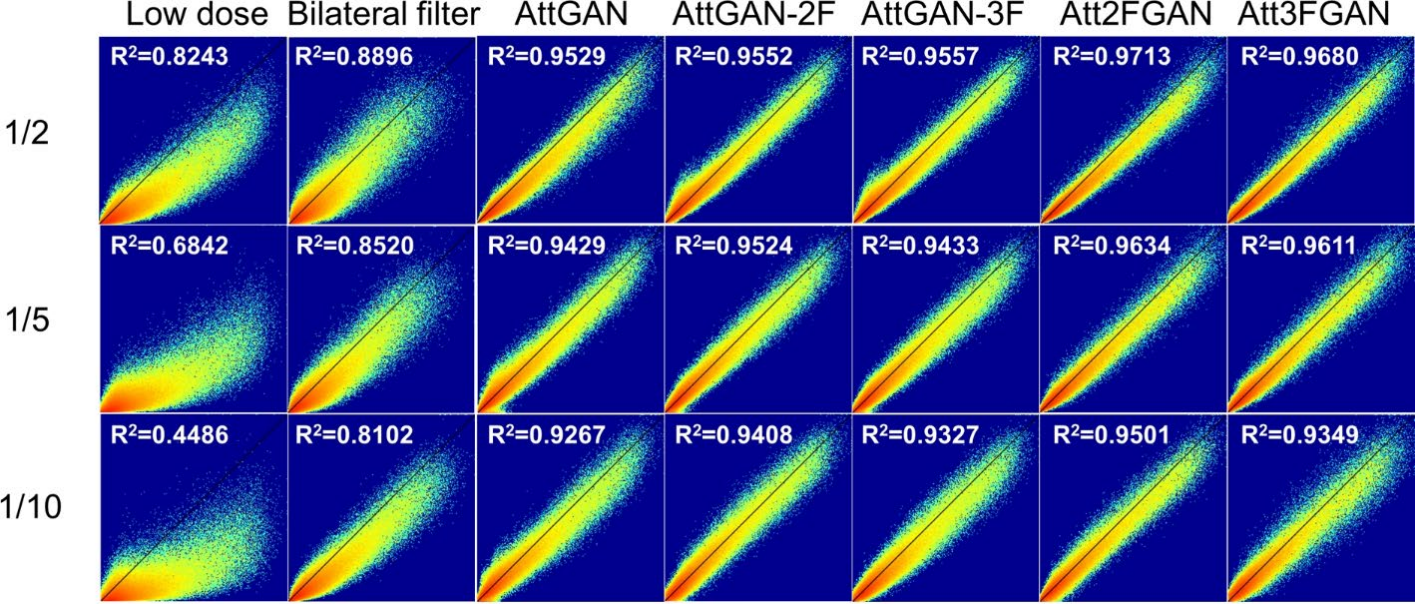
Joint histogram and linear regression results on the cardiac VOI of LD and DL-denoised SPECT images, using filtered FD SPECT images as reference

The joint correlation and linear regression results are displayed in Fig. 3. The results are generally similar to the previous quantitative indices. AttMFGAN demonstrates the best joint histogram and linear regression results with narrowest voxel count distributions as compared to those of FD and highest R^2^ values. Att2FGAN results are superior to Att3FGAN. The average NMSE and SSIM versus background noise expressed as CoV for various denoising methods on 1/10 LD SPECT images of 50 patients are shown in Supplementary Figure S1 (b) and (c). The Att2FGAN shows consistently superior noise and quantitative performance than AttGAN-2F, followed by AttGAN and LD SPECT (See Fig. 4).

The NMSE, SSIM and PSNR of 10 tested patients processed by Att2FGAN with global and local MAE loss, use only local (Att2FGAN-L) or global (Att2FGAN-G) MAE loss are shown in Table 3. The results of Att2FGAN-L and Att2FGAN-G were worse than Att2FGAN and AttGAN-2 F.

**Table 3:** The NMSE, SSIM and PSNR measurements (MEAN (95% CI)) on the cardiac VOI using AttGAN-2 F, Att2FGAN, Att2FGAN-L and Att2FGAN-G on 1/10 LD SPECT of 10 tested patients. (↓: lower value is better, ↑: higher value is better)

Metric	NMSE ↓	SSIM ↑	PSNR ↑
AttGAN-2 F	0.0602 (0.0462, 0.0742)	0.7877 (0.7541, 0.8213)	23.34 (21.84, 24.84)
Att2FGAN	0.0438 (0.0265, 0.0611)	0.8113 (0.7885, 0.8341)	25.02 (23.19, 26.85)
Att2FGAN-L	0.0652 (0.0490, 0.0814)	0.7765 (0.7384, 0.8146)	23.09 (21.57, 24.61)
Att2FGAN-G	0.0841 (0.0753, 0.0930)	0.7237 (0.6979, 0.7495)	21.77 (20.76, 22.78)

## Discussion

AttGAN is used as the baseline on denoising the projection-domain in this study based on our previous experience, i.e., the GAN is superior to CNN [20], attention block further improves denoising on GAN [18], and denoising on projection-domain is better than reconstruction-domain [17]. The LD MP SPECT projections are obtained by reducing the acquisition time of each view in projections instead of reducing view numbers as suggested by Shiri et al. [28] To the best of our knowledge, we firstly propose using multi-frequency denoising for LD MP SPECT, demonstrating superior performance as compared to our previous works [16–18, 20]. For example, Att2FGAN has better NMSE, SSIM, PSNR and similar PDS errors on 1/10 LD as compared to conventional AttGAN on 1/5 LD images. This denoising scheme can potentially be applied on denoising on reconstruction-domain as well as other DL denoising approaches.

From different quantitative comparisons, including various physical and clinical indices, the multi-frequency methods (AttGAN-MF and AttMFGAN) are consistently better than conventional DL methods (AttGAN). The neural network could learn the denoising task more efficiently by separating the original images into different frequency bands (Supplementary Figure S2), possibly similar to that fact that the human visual system acts as frequency-selective channels [29]. Denoising in respective frequency bands simplify the task, i.e., various frequency components of the images are then not superimposed. However, the multi-frequency denoising requires more processing time than mixed-frequency methods, e.g., AttGAN-2 F and Att2FGAN needs to train two different networks or generators, which doubles the training time (from ~ 2 h to ~ 4.5 h) and testing time (from ~ 0.1 s to ~ 0.25 s) as compared to AttGAN. Two frequency bands are generally better than three frequency bands for both multi-frequency denoising schemes, which could be attributed to the noise in higher frequency bands (*r* ≥ 20 pixels) were more challenging to ameliorate for the networks.

The radius of the frequency radial masks to separate low- and high-frequency is based on the visual assessment from the magnitude images of FD projections in the frequency domain. The circle region covering ~ 90% of the highest intensity in the center is selected as low frequency component. The radius to separate mid- and high- frequency is set to be double of that of the low frequency mask. We have preliminarily optimized the radius for AttGAN-2 F and find the use of 10-voxel radius is superior to 3, 5, 7, 15, and 20 -voxel radius for low- and high-frequency separation, thus is further used in this study. The optimal radius could be different for different applications with frequency distributions.

The proposed AttMFGAN further improves denoising performance as compared to AttGAN-MF. AttMFGAN consists of multiple generators for “local” multi-frequency projection images denoising and one discriminator for “global” evaluation based on the final denoised projection. The MAE between the final denoised projection and reference FD projection was added to the loss function of all generators as a global guidance for the training process, potentially leading to its superior performance, as indicated in our ablation study. Besides, the Fourier transform process would introduce negative values to different frequency bands of projection images. This could introduce negative values to final denoised projection for AttGAN-MF and AttMFGAN, but there are fewer negative values observed in the denoised projections on AttMFGAN (2/50 for Att2FGAN) as compared to AttGAN-MF (50/50 for AttGAN-2 F). Though all negative voxels have been set to zero before reconstructions, the denoising performance may still be compromised.

In addition, the AttGAN-MF and AttMFGAN with M = 2 are generally better than these with M = 3, which means the multi-frequency denoising dose not benefit from the separation of middle frequency and high frequency components. The possible reason could be that the high frequency components carry little useful information of the image. Learning useful information in this band could be challenging as most of the high frequency image is noise, leading to inferior performance of the networks. To verify our hypothesis, we have excluded the high frequency component in Att3FGAN (Att3FGAN-LM) in an ablation study. Our results show better NMSE and PSNR can be achieved from Att3FGAN-LM than the original Att3FGAN with high frequency generator (Supplementary Table S2). Thus, further separation of high frequency components may not help the multi-frequency denoising.

There are certain limitations in this study. The NMSE/SSIM versus noise trade-off curves were assessed to compare different methods rather than recovery coefficient versus noise analysis, due to the unavailability of ground truth in the clinical data, which could be potentially assessed by a phantom study with known ground truth [30, 31]. The PDS was evaluated as the only clinical-relevant analysis. Pretorius et al. [32] recently conducted a ROC study for LD MP SPECT denoising and found that convolutional autoencoder (CAE) did not significantly increase the area under the ROC curve (AUC) as compared to Gaussian filtered images in 1/4 and 1/8 LD levels. Though our previous study [20] demonstrated the superiority of 3D AttGAN to cGAN and to 3D CAE, further task-based analysis and ROC study are warranted to demonstrate the clinical effectiveness of the proposed multi-frequency denoising strategy using more clinical data.

## Conclusion

In this study, we proposed, implemented and evaluated multi-frequency AttMFGAN and AttGAN-MF for LD MP SPECT denoising. Multi-frequency denoising outperformed conventional AttGAN on multiple dose levels based on physical and clinical analysis, pushing the achievable LD limit for DL-based denoising.

## Electronic supplementary material

Below is the link to the electronic supplementary material.

Supplementary Material 1

## Data Availability

Authors will share data upon request to the corresponding author.

## Abbreviations

AC: Attenuation correction
AttGAN: Attention-guided conditional generative adversarial network
AttGAN-MF: Attention-guided conditional generative adversarial network with multiple frequency components
AttGAN-2F: Attention-guided conditional generative adversarial network with 2 frequency bands
AttGAN-3F: Attention-guided conditional generative adversarial network with 3 frequency bands
AttMFGAN: Attention-guided multi-frequency generative adversarial network
Att2FGAN: Attention-guided 2-frequency bands generative adversarial network
Att2FGAN-L: Attention-guided 2-frequency bands generative adversarial network with local generator loss
Att2FGAN-G: Attention-guided 2-frequency bands generative adversarial network with global generator loss
Att3FGAN: Attention-guided 3-frequency bands generative adversarial network
Att3FGAN-LM: Attention-guided 3-frequency bands generative adversarial network using only low- and mid-frequency bands
AUC: Area under the ROC curve
BCE: Binary cross-entropy error
BMI: Body mass index
CAE: Convolutional auto-encoder
cGAN: Conditional generative adversarial network
CNN: Convolutional neural network
CoV: Coefficient of variance
CT: Computed tomography
DL: Deep learning
FD: Full dose
FFT: Fast Fourier transform
HLA: Horizontal long axis
LD: Low dose
MAE: Mean absolute error
MF: Multi-frequency
MP SPECT: Myocardial perfusion single photon emission computed tomography
NMSE: Normalized mean absolute error
OS-EM: Ordered subset-expectation maximization
PDS: Perfusion defect size
PSNR: Peak signal-to-noise ratio
ROC: Receiver-operating-characteristic
SA: Short axis
SSIM: Structural similarity
VOI: Volume-of-interest
