# Supplementary material for: Deep learning-based multi-frequency denoising for myocardial perfusion SPECT
Source: EJNMMI Phys. 2024 Oct 2;11:80. doi: 10.1186/s40658-024-00680-w (PMC11447183; doi:10.1186/s40658-024-00680-w)
Supplement: Supplementary file 1 — Supplementary Material 1 [file 40658_2024_680_MOESM1_ESM.docx]

**Deep learning-based multi-frequency denoising for myocardial perfusion SPECT**

Table S1. Average NMSE on whole reconstructed SPECT of 50 patients denoised by bilateral filter with different parameters of pixel neighborhood (***N***), filter sigma in the spatial (***σ_d_***) and intensity (***σ_r_***) distance. Bold values indicate best results in each choice of ***N***.

| ***N***=1,2,3 | | | | | | | | | | |
| --- | --- | --- | --- | --- | --- | --- | --- | --- | --- | --- |
| ***σ_r_***  ***σ_d_*** | 1 | 2 | 3 | 4 | 5 | 6 | 7 | 8 | 9 | 10 |
| 1 | 0.1835 | 0.1835 | 0.1835 | 0.1835 | 0.1835 | 0.1835 | 0.1835 | 0.1835 | 0.1835 | 0.1835 |
| 2 | 0.1749 | 0.1749 | 0.1749 | 0.1749 | 0.1749 | 0.1749 | 0.1749 | 0.1749 | 0.1749 | 0.1749 |
| 3 | 0.1662 | 0.1661 | 0.1661 | 0.1661 | 0.1661 | 0.1661 | 0.1661 | 0.1661 | 0.1661 | 0.1661 |
| 4 | 0.1585 | 0.1585 | 0.1585 | 0.1585 | 0.1585 | 0.1585 | 0.1585 | 0.1585 | 0.1585 | 0.1585 |
| 5 | 0.1521 | 0.1521 | 0.1521 | 0.1521 | 0.1521 | 0.1521 | 0.1520 | 0.1520 | 0.1520 | 0.1520 |
| 6 | 0.1465 | 0.1464 | 0.1464 | 0.1464 | 0.1464 | 0.1464 | 0.1464 | 0.1464 | 0.1464 | 0.1464 |
| 7 | 0.1292 | 0.1292 | **0.1291** | 0.1291 | 0.1291 | 0.1291 | 0.1291 | 0.1291 | 0.1291 | 0.1291 |
| 8 | 0.1329 | 0.1329 | 0.1328 | 0.1328 | 0.1328 | 0.1328 | 0.1328 | 0.1328 | 0.1328 | 0.1328 |
| 9 | 0.1370 | 0.1369 | 0.1369 | 0.1369 | 0.1369 | 0.1369 | 0.1369 | 0.1369 | 0.1369 | 0.1369 |
| 10 | 0.1415 | 0.1414 | 0.1414 | 0.1414 | 0.1414 | 0.1414 | 0.1414 | 0.1414 | 0.1414 | 0.1414 |
| ***N***=4,5 | | | | | | | | | | |
| ***σ_r_***  ***σ_d_*** | 1 | 2 | 3 | 4 | 5 | 6 | 7 | 8 | 9 | 10 |
| 1 | 0.1827 | 0.1827 | 0.1827 | 0.1827 | 0.1827 | 0.1827 | 0.1827 | 0.1827 | 0.1827 | 0.1827 |
| 2 | 0.1717 | 0.1717 | 0.1717 | 0.1717 | 0.1717 | 0.1717 | 0.1717 | 0.1717 | 0.1717 | 0.1717 |
| 3 | 0.1614 | 0.1614 | 0.1614 | 0.1614 | 0.1614 | 0.1614 | 0.1614 | 0.1614 | 0.1614 | 0.1614 |
| 4 | 0.1529 | 0.1529 | 0.1529 | 0.1529 | 0.1529 | 0.1529 | 0.1529 | 0.1529 | 0.1529 | 0.1529 |
| 5 | 0.1459 | 0.1459 | 0.1459 | 0.1459 | 0.1459 | 0.1459 | 0.1459 | 0.1459 | 0.1459 | 0.1459 |
| 6 | 0.1398 | 0.1398 | 0.1398 | 0.1398 | 0.1398 | 0.1398 | 0.1398 | 0.1398 | 0.1398 | 0.1398 |
| 7 | 0.1345 | 0.1344 | 0.1344 | 0.1344 | 0.1344 | 0.1344 | 0.1344 | 0.1344 | 0.1344 | 0.1344 |
| 8 | 0.1201 | **0.1200** | 0.1200 | 0.1200 | 0.1200 | 0.1200 | 0.1200 | 0.1200 | 0.1200 | 0.1200 |
| 9 | 0.1247 | 0.1247 | 0.1246 | 0.1246 | 0.1246 | 0.1246 | 0.1246 | 0.1246 | 0.1246 | 0.1246 |
| 10 | 0.1295 | 0.1294 | 0.1294 | 0.1294 | 0.1294 | 0.1294 | 0.1294 | 0.1294 | 0.1294 | 0.1294 |
| ***N***=6 | | | | | | | | | | |
| ***σ_r_***  ***σ_d_*** | 1 | 2 | 3 | 4 | 5 | 6 | 7 | 8 | 9 | 10 |
| 1 | 0.1825 | 0.1825 | 0.1825 | 0.1825 | 0.1825 | 0.1825 | 0.1825 | 0.1825 | 0.1825 | 0.1825 |
| 2 | 0.1718 | 0.1718 | 0.1718 | 0.1718 | 0.1718 | 0.1718 | 0.1718 | 0.1718 | 0.1718 | 0.1718 |
| 3 | 0.1626 | 0.1627 | 0.1627 | 0.1627 | 0.1627 | 0.1627 | 0.1627 | 0.1627 | 0.1627 | 0.1627 |
| 4 | 0.1556 | 0.1556 | 0.1556 | 0.1556 | 0.1556 | 0.1556 | 0.1556 | 0.1556 | 0.1556 | 0.1556 |
| 5 | 0.1500 | 0.1500 | 0.1501 | 0.1501 | 0.1501 | 0.1501 | 0.1501 | 0.1501 | 0.1501 | 0.1501 |
| 6 | 0.1453 | 0.1454 | 0.1455 | 0.1455 | 0.1455 | 0.1455 | 0.1455 | 0.1455 | 0.1455 | 0.1455 |
| 7 | 0.1337 | 0.1338 | 0.1339 | 0.1339 | 0.1339 | 0.1339 | 0.1339 | 0.1339 | 0.1339 | 0.1339 |
| 8 | 0.1300 | **0.1299** | 0.1299 | 0.1299 | 0.1299 | 0.1299 | 0.1299 | 0.1299 | 0.1300 | 0.1300 |
| 9 | 0.1378 | 0.1377 | 0.1377 | 0.1377 | 0.1377 | 0.1377 | 0.1377 | 0.1377 | 0.1377 | 0.1377 |
| 10 | 0.1414 | 0.1414 | 0.1415 | 0.1415 | 0.1415 | 0.1415 | 0.1415 | 0.1415 | 0.1415 | 0.1415 |
| ***N***=7 | | | | | | | | | | |
| ***σ_r_***  ***σ_d_*** | 1 | 2 | 3 | 4 | 5 | 6 | 7 | 8 | 9 | 10 |
| 1 | 0.1828 | 0.1828 | 0.1828 | 0.1828 | 0.1828 | 0.1828 | 0.1828 | 0.1828 | 0.1828 | 0.1828 |
| 2 | 0.1733 | 0.1734 | 0.1734 | 0.1734 | 0.1734 | 0.1734 | 0.1734 | 0.1734 | 0.1734 | 0.1734 |
| 3 | 0.1660 | 0.1661 | 0.1661 | 0.1661 | 0.1661 | 0.1661 | 0.1661 | 0.1661 | 0.1661 | 0.1661 |
| 4 | 0.1609 | 0.1610 | 0.1610 | 0.1611 | 0.1611 | 0.1611 | 0.1611 | 0.1611 | 0.1611 | 0.1611 |
| 5 | 0.1572 | 0.1574 | 0.1574 | 0.1574 | 0.1574 | 0.1574 | 0.1574 | 0.1575 | 0.1575 | 0.1575 |
| 6 | 0.1543 | 0.1546 | 0.1547 | 0.1547 | 0.1547 | 0.1547 | 0.1547 | 0.1547 | 0.1547 | 0.1547 |
| 7 | 0.1520 | 0.1523 | 0.1524 | 0.1524 | 0.1524 | 0.1524 | 0.1524 | 0.1524 | 0.1524 | 0.1524 |
| 8 | 0.1455 | 0.1451 | **0.1450** | 0.1451 | 0.1451 | 0.1451 | 0.1451 | 0.1451 | 0.1451 | 0.1451 |
| 9 | 0.1476 | 0.1476 | 0.1477 | 0.1477 | 0.1478 | 0.1478 | 0.1478 | 0.1478 | 0.1478 | 0.1478 |
| 10 | 0.1497 | 0.1501 | 0.1501 | 0.1502 | 0.1502 | 0.1502 | 0.1502 | 0.1502 | 0.1502 | 0.1502 |


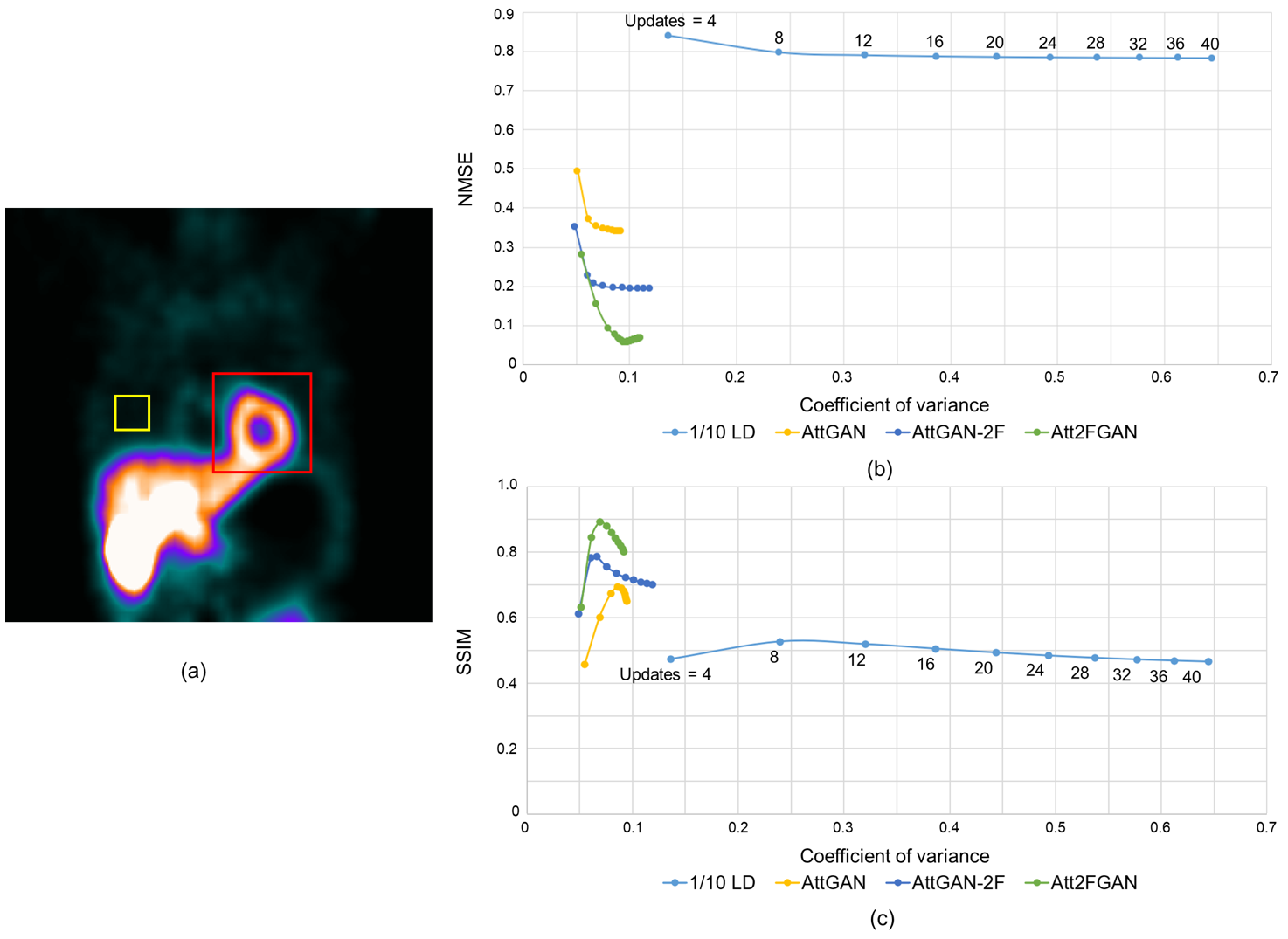


Figure S1. (a) Sample VOIs for coefficient of variance (CoV, yellow box), NMSE and SSIM measurement (red box), (b) Average NMSE versus background CoV, and (c) Average SSIM versus background CoV for various denoising methods on 1/10 LD SPECT images of 50 patients.


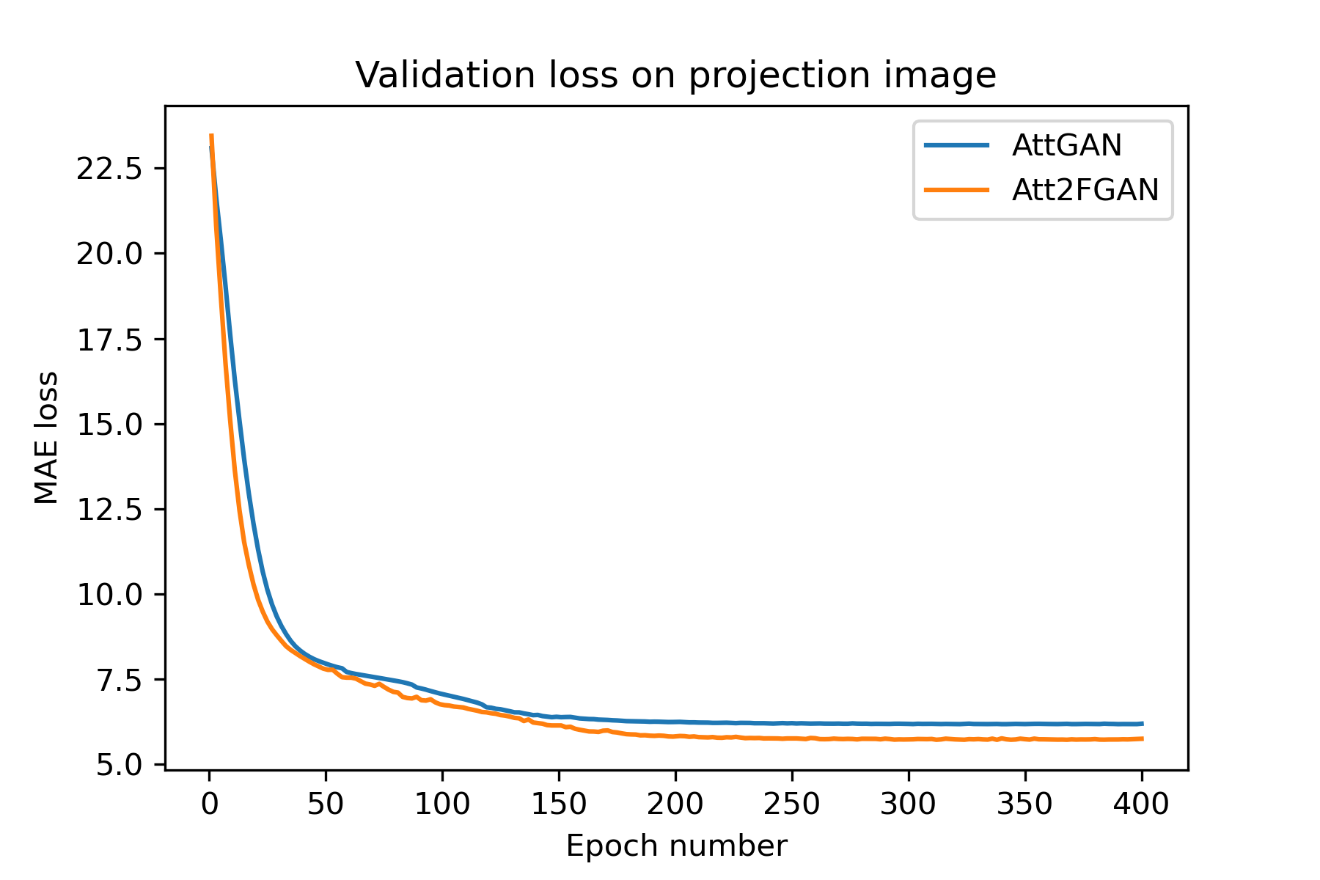


Figure S2. Validation MAE loss versus training epochs curves of AttGAN and Att2FGAN.

Table S2. The NMSE, SSIM and PSNR measurements (MEAN (95% CI)) on the cardiac VOI using Att2FGAN, Att3FGAN-LM, and Att3FGAN on 1/10 LD SPECT of 10 tested patients. (↓: lower value is better, ↑: higher value is better)

| **Metric** | **NMSE ↓** | **SSIM ↑** | **PSNR ↑** |
| --- | --- | --- | --- |
| **Att2FGAN** | 0.0438 (0.0265, 0.0611) | 0.8113 (0.7885, 0.8341) | 25.02 (23.19, 26.85) |
| **Att3FGAN-LM** | 0.0583 (0.0449, 0.0717) | 0.7751 (0.7288, 0.8214) | 24.44 (22.97, 25.91) |
| **Att3FGAN** | 0.0662 (0.0488, 0.0836) | 0.7893 (0.7431, 0.8355) | 23.93 (22.26, 25.60) |
